# Supplementary material for: Synthesis and Antiproliferative Activity against Cancer Cells of Indole-Aryl-Amide Derivatives
Source: Molecules. 2022 Dec 28;28(1):265. doi: 10.3390/molecules28010265 (PMC9822155; doi:10.3390/molecules28010265)
Supplement: Supplementary file 1 [file molecules-28-00265-s001.zip › molecules-2082321-supplementary.pdf]

Supporting Information

Synthesis and Antiproliferative activity against cancer cells of Indole-Aryl-Amide Derivatives

Junwei Zhao,[1] Jacopo Carbone,[1] Giovanna Farruggia[2], Anna Janecka,[3] Luca Gentilucci,[1,] * Natalia Calonghi.[2,] *

[1] Department of Chemistry "G. Ciamician", University of Bologna, Via Selmi 2, 40126-Bologna, Italy; juwzhao@ucdavis.edu (J.Z.), jacopo.carbone2@studio.unibo.it (J.C.)
[2] Department of Pharmacy and Biotechnology (FaBiT), University of Bologna, Via Irnerio 48, 40126 Bologna, Italy; giovanna.farruggia@unibo.it (GF)
[3] Department of Biomolecular Chemistry, Medical University of Lodz, Mazowiecka 6/8, 92-215 Lodz, Poland; anna.janecka@umed.lodz.pl (AJ).
* Correspondence: luca.gentilucci@unibo.it (L.G.); natalia.calonghi@unibo.it (N.C.);
Tel: +39-05-1209-9570 (L.G.); +39-05-1209-1231 (N.C.)

Table of Contents

Figures S1–S7. ^{1}H NMR of **1–7** in $CDCl_3$, 400 MHz. ……………………………………………… pS2

Figure S8. Alternative views of the binding poses of **2** and **5** in KOR ………………………... pS5

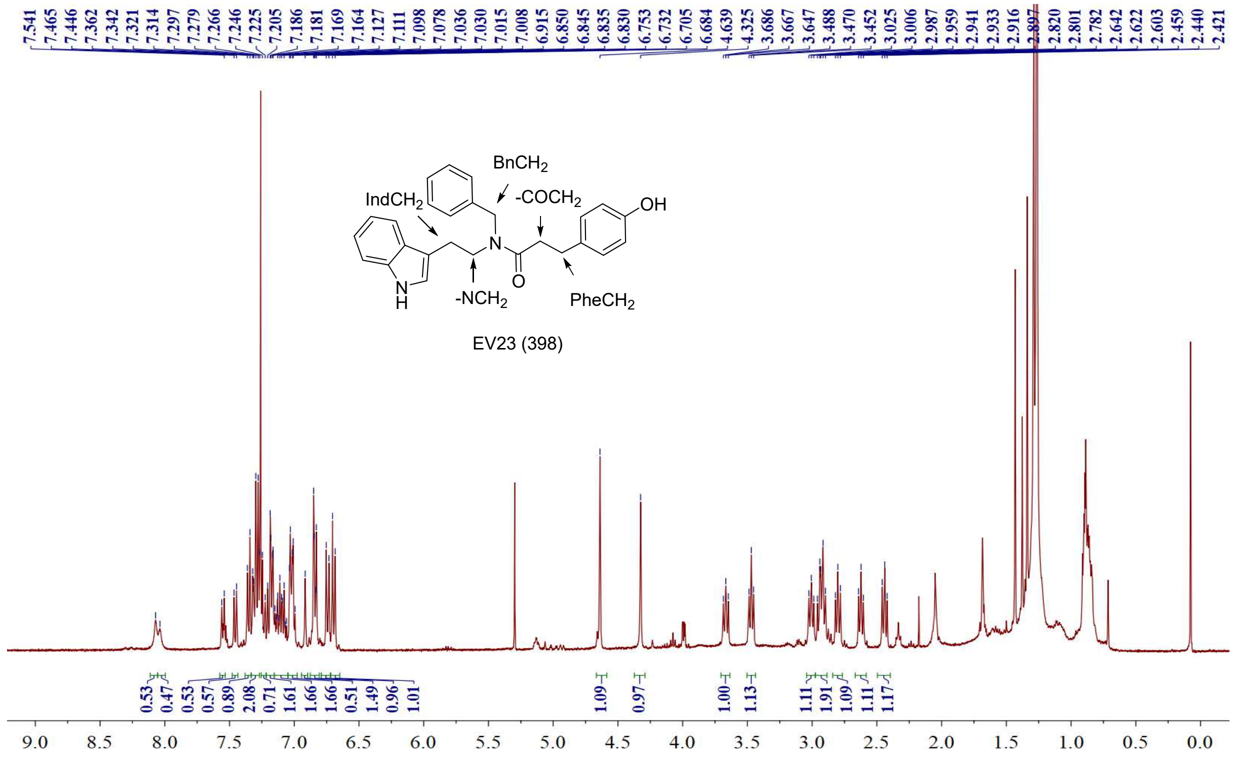

Figure S1. 1H NMR of **1** in CDCl3, 400 MHz.

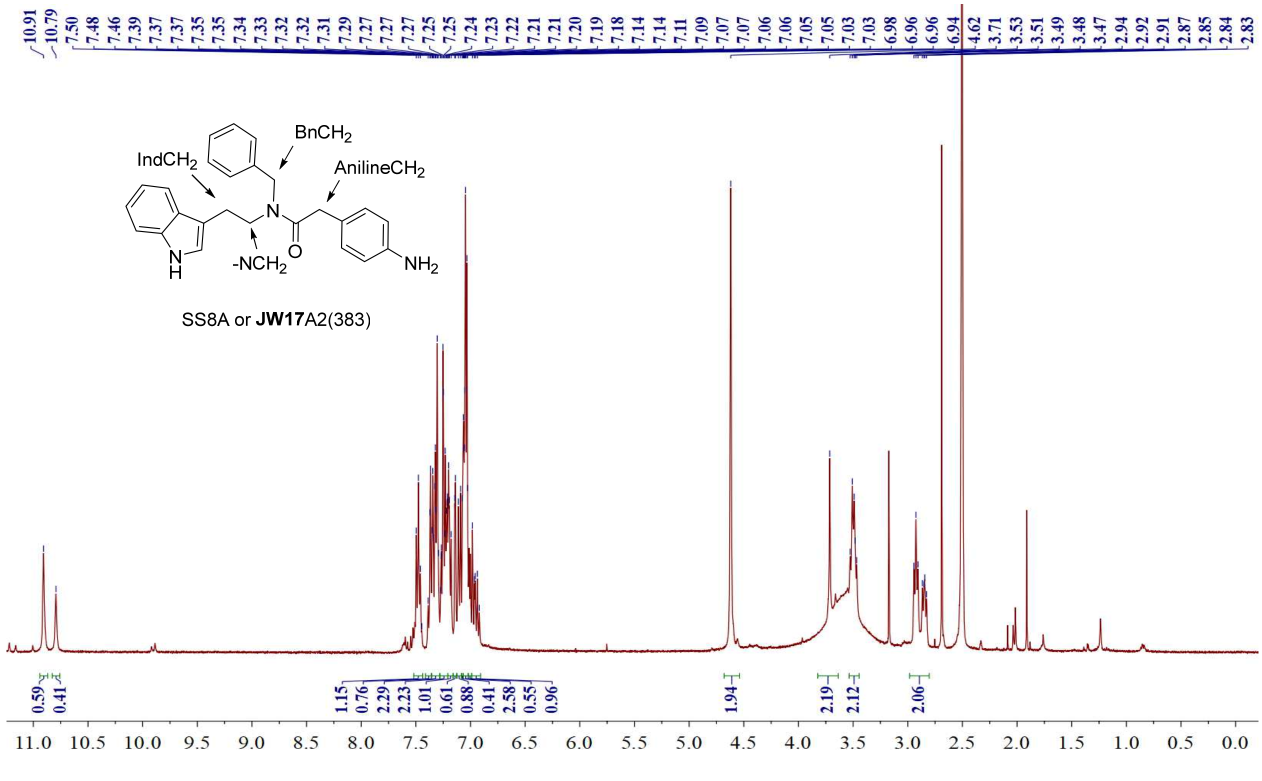

Figure S2. 1H NMR of **2 i**n CDCl3, 400 MHz.

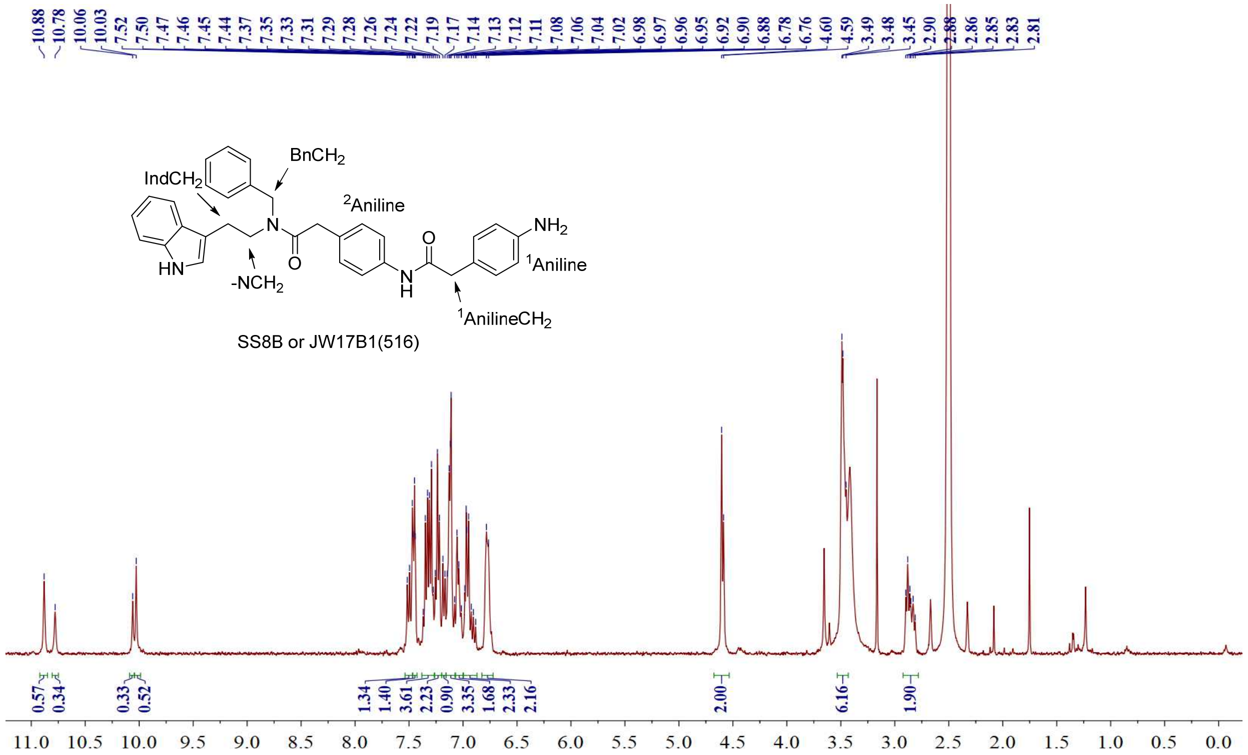

Figure S3. 1H NMR of **3 i**n CDCl3, 400 MHz.

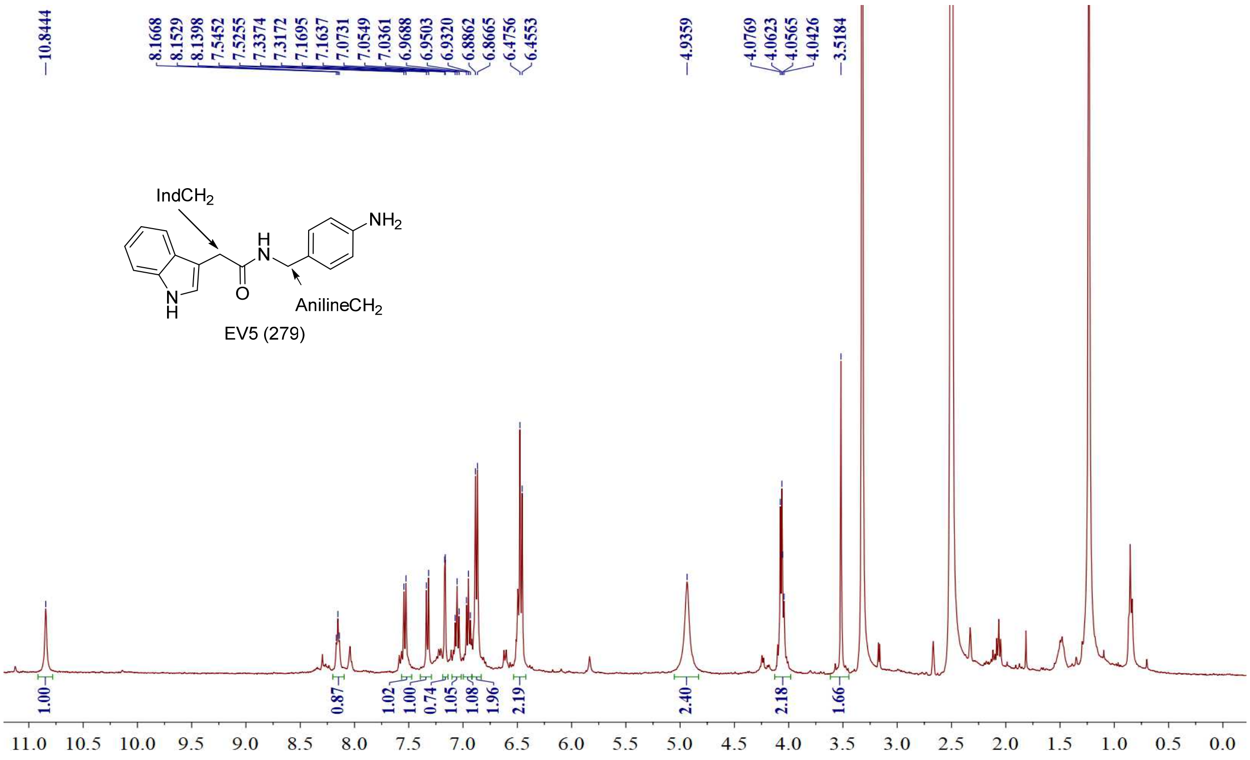

Figure S4. 1H NMR of **4 i**n CDCl3, 400 MHz.

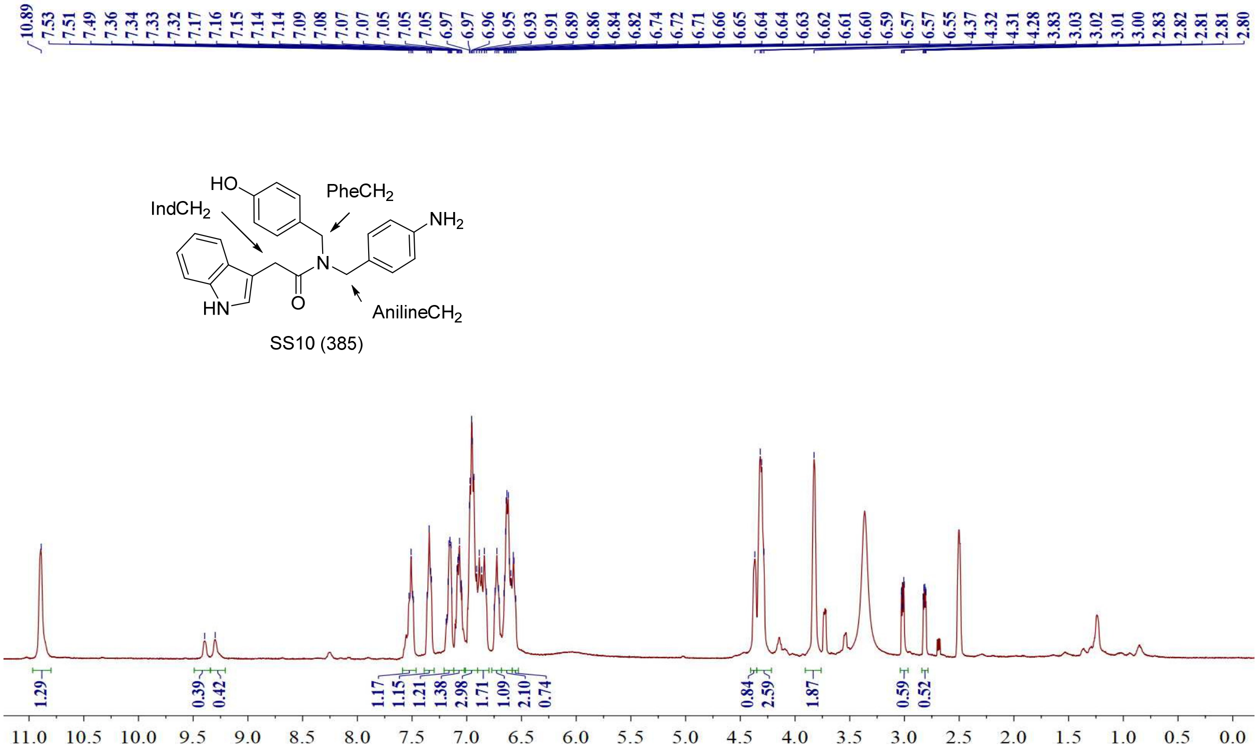

Figure S5. 1H NMR of **5 i**n CDCl3, 400 MHz.

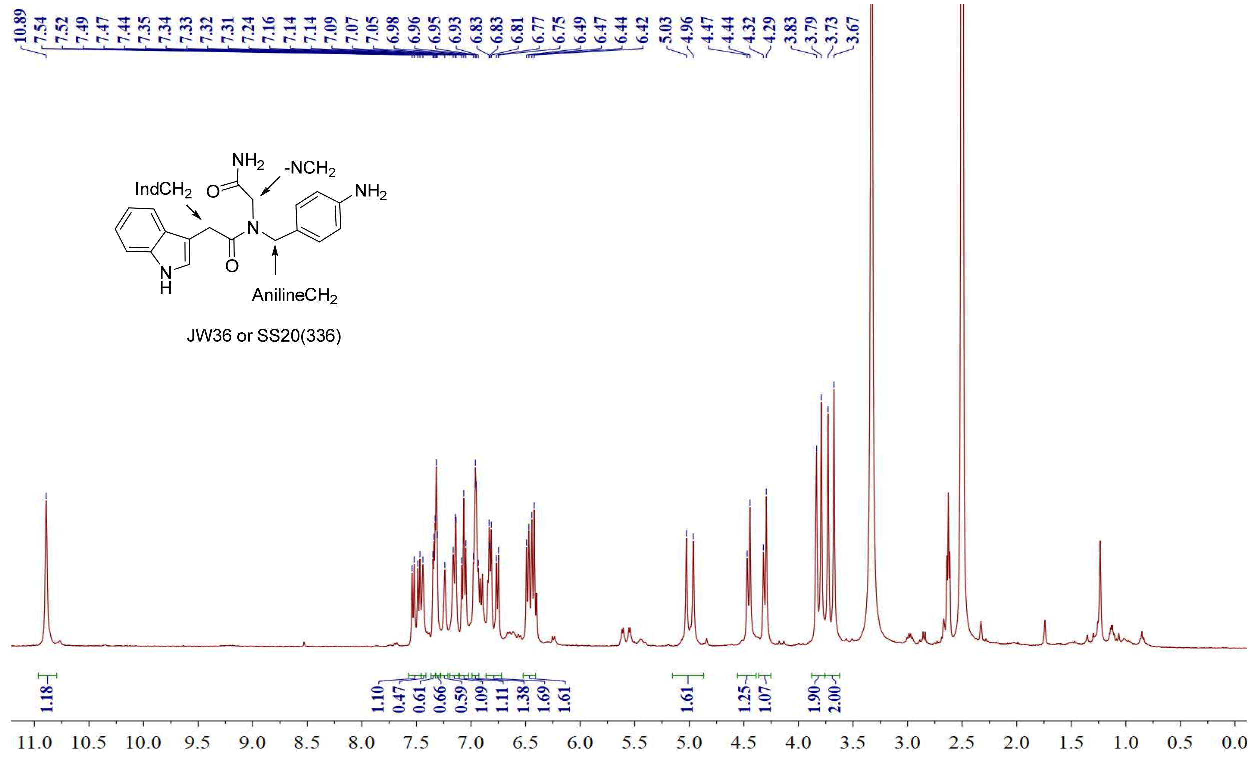

Figure S6. 1H NMR of **6 i**n CDCl3, 400 MHz.

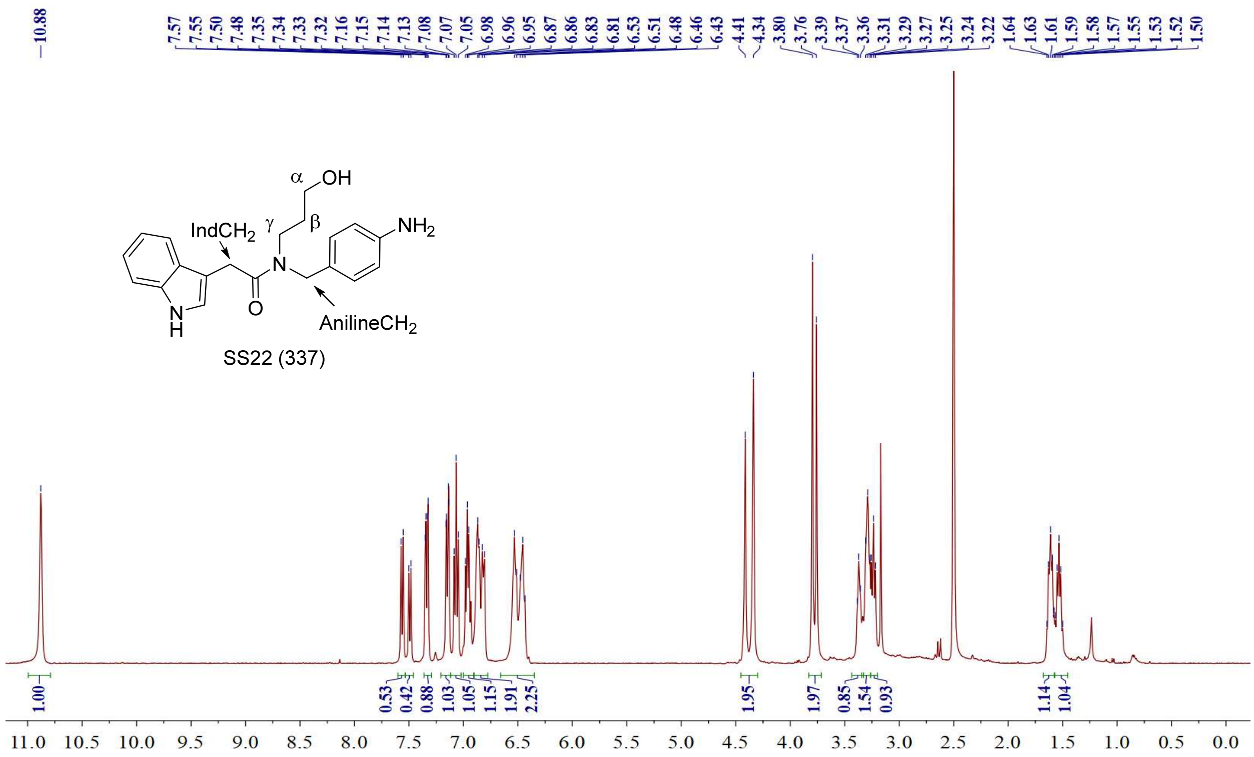

Figure S7. 1H NMR of **7 i**n CDCl3, 400 MHz.

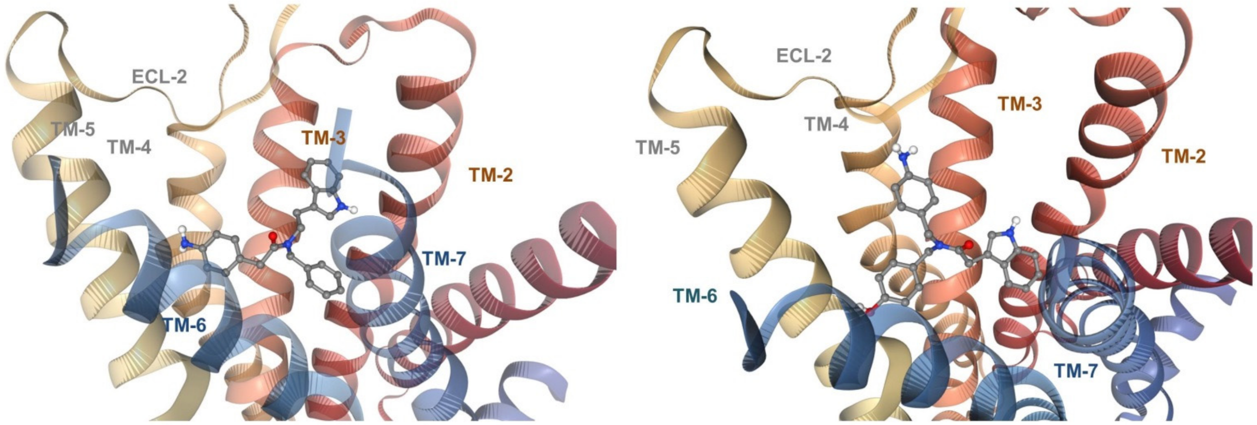

Figure S8. Top views of the predicted binding poses of **2** (left) and **5** (right) in KOR. The ligands are rendered in thick lines; C is rendered in grey, N in blue, and O in red. Figure obtained with PacDOCK web server (https://pegasus.lbic.unibo.it/pacdock/)
